# Supplementary material for: Detection of Feline Coronavirus RNA in Cats with Feline Infectious Peritonitis and Their Housemates
Source: Viruses. 2025 Jul 4;17(7):948. doi: 10.3390/v17070948 (PMC12300432; doi:10.3390/v17070948)
Supplement: Supplementary file 1 [file viruses-17-00948-s001.zip › viruses-3648257-supplementary.pdf]

SUPPLEMENTARY MATERIAL

Detection of Feline Coronavirus RNA in Cats with Feline Infectious Peritonitis and Their Housemates

Phoenix M. Shepherd [1,2,3], Amy Elbe [4], Brianna M. Lynch [4], Erin Lashnits [4] and Robert N. Kirchdoerfer [1,2,3,*]

[1] Biochemistry Department, University of Wisconsin-Madison, Madison, WI 53706, USA
[2] Institute for Molecular Virology, University of Wisconsin-Madison, Madison, WI 53706, USA
[3] Center for Quantitative Cell Imaging, University of Wisconsin-Madison, Madison, WI 53706, USA
[4] Department of Medical Sciences, School of Veterinary Medicine, University of Wisconsin-Madison, Madison, WI 53706, USA

* Correspondence: rnkirchdoerf@wisc.edu

Contents:

Supplementary Table S1
Supplementary Figures S1–S3

	Control Cat 001	Control Cat 002	Control Cat 003	Control Cat 004	Control Cat 005	Control Cat 006	Control Cat 007	Control Cat 008	Control Cat 009
Plasma	Below LoD	Below LoD	Below LoD	Below LoD	Below LoD	Below LoD	Below LoD	NA	Below LoD
Whole Blood	Below LoD	Below LoD	1.7e5 RNA copies/ml	Below LoD	Below LoD	Below LoD	Detected	Below LoD	Below LoD
Fecal Swabs	Detected	Below LoD	2.9e6 RNA copies/swab	Below LoD	Below LoD	5.6e7 RNA copies/swab	Below LoD	Below LoD	Below LoD
Conunctival Swabs	6.5e6 RNA copies/swab	6.0e6 RNA copies/swab	Detected	Detected	Detected	Detected	Detected	Detected	Detected

Supplemental Table 1: qRT-PCR results from nine unexposed, healthy cats. Quantifiable samples have numerical values. LoD, limit of detection. NA, not applicable due to low sample availability. Samples testing below the limit of quantification but above the limit of detection are labelled as “Detected”.

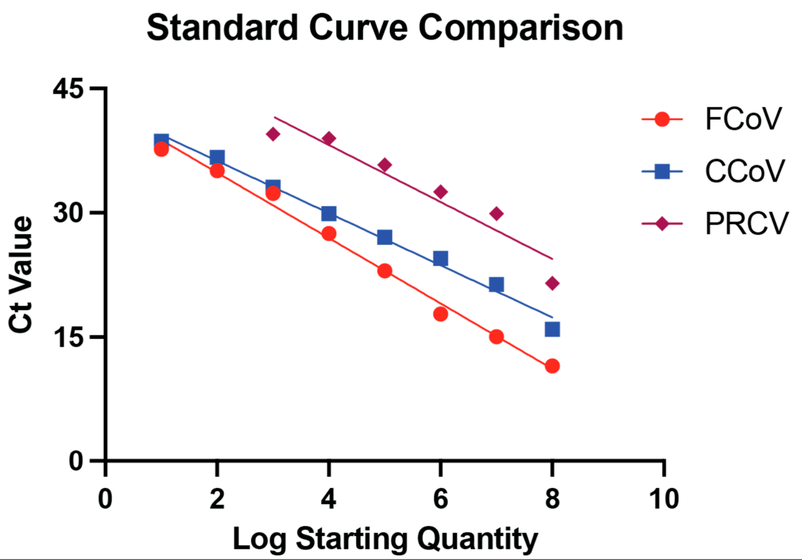

Supplemental Figure 1: Assay cross-reactivity with similar Alphacoronaviruses. Standard curves ranging from 1.0E8 to 1.0E1 were generated for three coronaviruses: Feline (FCoV, red), Canine (CCoV, blue), and Porcine (PRCV, purple). Graph displays decreased assay sensitivity for CCoV at higher amount of standard, but comparable levels at lower amounts compared to FCoV standards (compare red to blue). Loss of sensitivity is greater when comparing PRCV and FCoV at all amounts tested (compared red to purple).

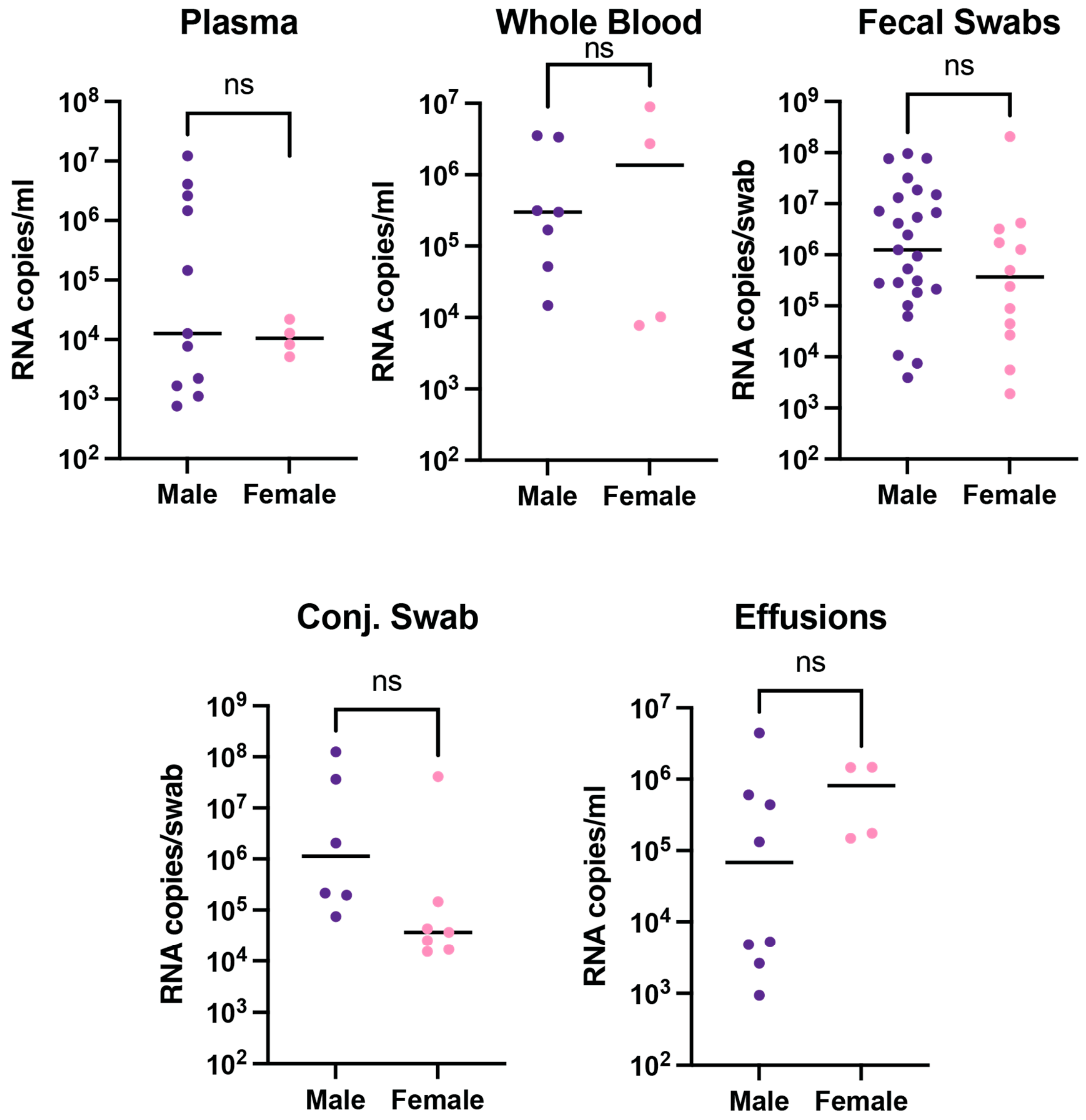

Supplemental Figure 2: Sex differences per sample type. Quantifiable samples were divided based on sex of the cat. Male and female cats were compared for each sample type, with no differences in RNA copies observed. ns, not significant.

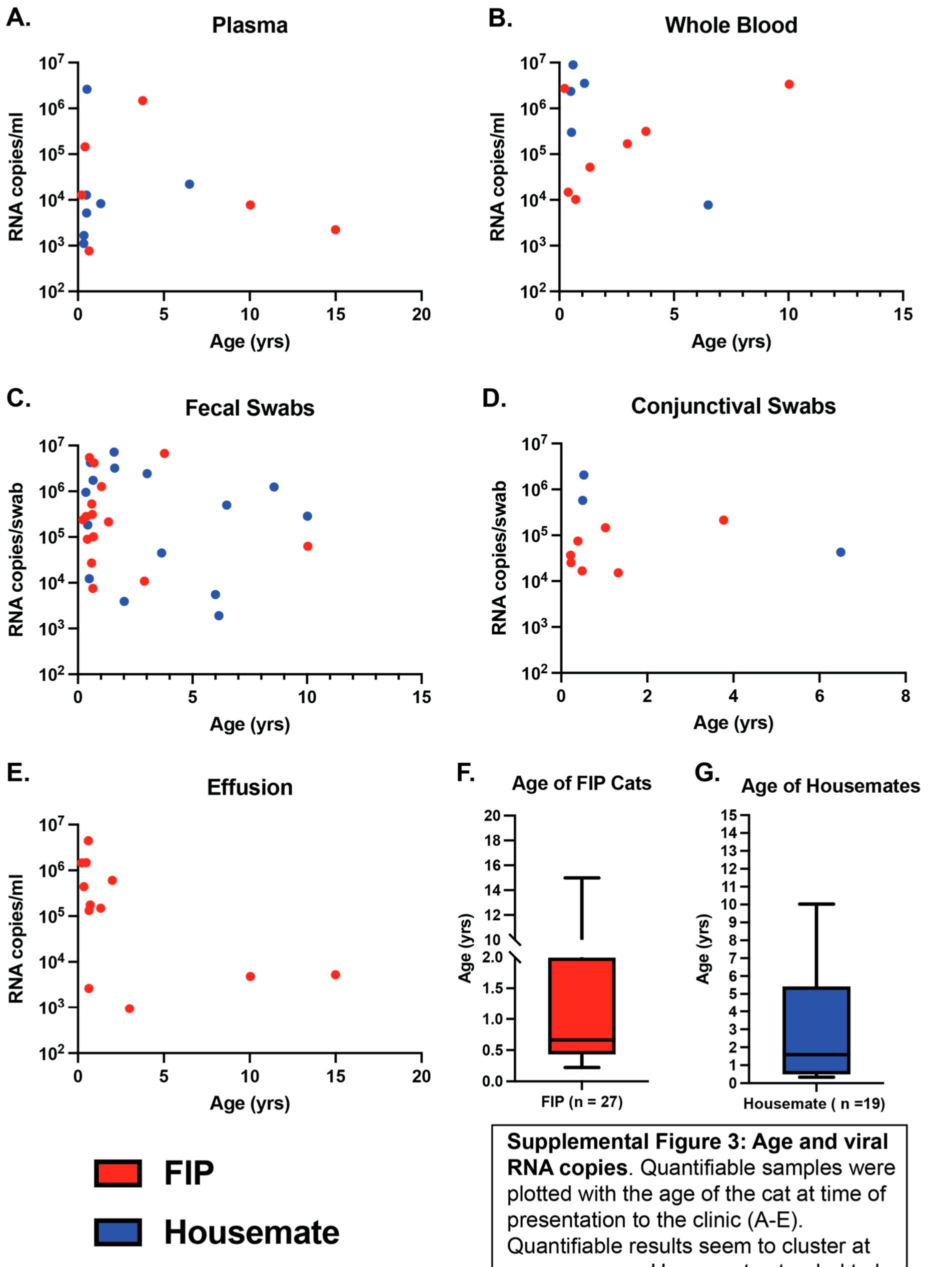

Supplemental Figure 3: Age and viral RNA copies. Quantifiable samples were plotted with the age of the cat at time of presentation to the clinic (A-E). Quantifiable results seem to cluster at younger ages. Housemates tended to be older (median age 1.59 years) than cats with FIP (median age 0.66 years, F-G).
